# Chemical Variability of the Essential Oils from Two Portuguese Apiaceae: *Coriandrum sativum* L. and *Foeniculum vulgare* Mill.

**DOI:** 10.3390/plants12142749

**Published:** 2023-07-24

**Authors:** Alexandra M. Machado, Violeta Lopes, Ana Maria Barata, Orlanda Póvoa, Noémia Farinha, Ana Cristina Figueiredo

**Affiliations:** 1 Centro de Estudos do Ambiente e do Mar (CESAM Lisboa), Faculdade de Ciências da Universidade de Lisboa (FCUL), Biotecnologia Vegetal, DBV, C2, Campo Grande, 1749-016 Lisboa, Portugal; 2 Banco Português de Germoplasma Vegetal (BPGV), Instituto Nacional de Investigação Agrária e Veterinária, Quinta de S. José, S. Pedro de Merelim, 4700-859 Braga, Portugal; violeta.lopes@iniav.pt (V.L.); anamaria.barata@iniav.pt (A.M.B.); 3 VALORIZA—Centro de Investigação para a Valorização de Recursos Endógenos, Instituto Politécnico de Portalegre, Praça do Município 11, 7300-110 Portalegre, Portugal; opovoa@ipportalegre.pt; 4 Instituto Politécnico de Portalegre, Praça do Município 11, 7300-110 Portalegre, Portugal; nfarinha@ipportalegre.pt

**Keywords:** Portuguese medicinal and aromatic plants, Apiaceae, essential oil composition, cluster analysis, chemotypes, chemical descriptors

## Abstract

*Coriandrum sativum* L. and *Foeniculum vulgare* Mill. are two aromatic and medicinal Apiaceae species commonly grown in Portugal, whose essential oils (EOs) are used in the food, pharmaceutical, and cosmetics industries. The present study evaluated EOs isolated from the fruits and vegetative aerial parts (VAPs) of 11 samples of *Coriandrum sativum* L. and from the fruits of 19 samples of *Foeniculum vulgare* Mill. The plant material was grown in experimental fields, after collection from several regions of mainland Portugal. The EOs were isolated by hydrodistillation and analyzed by gas chromatography and gas chromatography–mass spectrometry. The coriander EOs analysis evidenced two main clusters, with the first containing the fruits’ EOs dominated by linalool (60–73%), γ-terpinene, and α-pinene and the second with the VAPs’ EOs, which showed 2-*trans*-decenal (37–63%) and *n*-decanal (13–30%) as the main compounds. The fennel EOs analysis revealed two well correlated clusters, the first dominated by estragole (34–76%) and fenchone (16–30%) and the other dominated by *trans*-anethole (37–56%) and fenchone (14–34%). The present data suggest coriander EOs’ chemical descriptors as linalool for the fruits’ EOs and 2-*trans*-decenal with *n*-decanal for the VAPs’ EOs. For the fennel fruit EOs, the putative descriptors were *trans*-anethole and estragole, with variable fenchone content. The gathered data reinforce the relevance of clarifying variability of these species’ EOs, particularly when considering aromatic and medicinal plants with such a wide range of applications.

## 1. Introduction

Apiaceae or Umbelliferae, included in the Apiales order, is a family of dicotyledonous flowering plants comprising nearly 300–455 genera with around 3000–3750 species, distributed from the northern temperate regions to the high-altitude regions in tropical areas [1,2]. The largest number of genera in this family is located in Asia, 289, with Europe having about 126 and Africa having around 121 [3,4]. Apiaceae members exhibit a large number of phytochemical compounds, like flavonoids, coumarins, terpenoids, and polyacetylenes, as well as steroids, consisting of economically important aromatic plants, with applications in the food, pharmaceutical, perfumery, and cosmetics industries [5,6,7]. There are also reports of their application as repellents and in traditional medicine for the treatment of gastrointestinal, reproductive, and respiratory diseases [2,5].

Some economically valuable food plants, herbs, and spices of Apiaceae include *Anethum graveolens* L. (dill), *Apium graveolens* L. (celery), *Carum carvi* L. (caraway), *Centella asiatica* L. (Gotu kola), *Coriandrum sativum* L. (coriander), *Cuminum cyminum* L. (cumin), *Daucus carota* L. (carrot), *Foeniculum vulgare* Mill. (fennel), *Petroselinum crispum* (Mill.) Nyman ex A. W. Hill (parsley), and *Pimpinella anisum* L. (anise) [3,8].

Several species in this family are recognized for their particular aroma and are an excellent source of essential oils (EOs) obtained from the fruits, stems, leaves, and roots, with more than 760 different constituents of various chemical classes of significant pharmaceutical interest [2,5,9,10]. Several studies have shown that Apiaceae’s EOs possess a wide range of biological activities, such as antioxidant, antimicrobial, anti-inflammatory, anticarcinogenic, antidiabetic, cardioprotective, hypocholesterolemic, hepatoprotective, and neuroprotective properties [3,11,12,13,14,15,16,17,18,19,20,21].

The Apiaceae family is well represented on the Iberian Peninsula with several endemic species, with some of them included in the Portuguese flora [22,23].

Coriander (*Coriandrum sativum* L.), an herbaceous plant with aromatic fruits of 4 to 5 mm in diameter, is native to the Mediterranean region and is mainly cultivated in the temperate regions that surround the Mediterranean Basin, as well in Eastern Europe, India, China, and Thailand. Different plant parts are used for human consumption, like the stem and leaves as a vegetable or culinary herb, and the seed (fruit) and the seed oil are utilized as a spice and also in traditional medicine [1,5,24,25]. Dried seeds (fruits) have been used for approximately 7000 years, and their oil has been used as a food ingredient and fragrance since around 1900 [26]. Interestingly, from the seeds, both an oil (rich in fatty acids) and an essential oil (terpene rich) can be obtained, according to the extraction procedure, each of which have diverse applications. In 2014, the European Commission approved placing coriander seed oil (not the essential oil) on the market as a novel food ingredient according to Regulation (EC) No. 258/97 of the European Parliament and of the Council due to its richness in petroselinic acid (an isomer of oleic acid) [5,27]. *C. sativum* fruits are commonly known as coriander seeds, with the “coriander” word usually indicating the fruit not the plant [28].

Coriander leaves are usually called Chinese parsley, fresh coriander, “asotu” in Eastern Anatolia, or cilantro in the United States, with them being consumed fresh in several dishes. The aroma of coriander fruits differs completely from that of the leaves and/or the herb. The characteristic aerial parts’ odor (not consensual regarding pleasantness) is provided by aliphatic aldehydes, which constitute the main compounds of fresh herb EOs, while oxygen-containing monoterpenes like linalool as well as monoterpene hydrocarbons dominate fruit EOs [28,29].

European Pharmacopoeia 10 [30] as well as the Portuguese Pharmacopeia IX [31] include one monograph concerning *C. sativum* dried fruits, in which the EO content should be no less than 3 mL/kg. Both the European Pharmacopeia 10 [30] and ISO 3516:1997 [32] mention the EO main component range, namely 65–78% linalool.

Fennel (*Foeniculum vulgare* Mill.) is an herbaceous Apiaceae species, with finely divided leaves and umbels with yellow flowers and a characteristic odor. The genus has a wide distribution range across the Macaronesia region, the west and south of Europe, the north of Africa, and South-West and Central Asia. It occurs spontaneously in a wide area and is both cultivated and naturalized in the Mediterranean area. Traditionally, it has been treated as a single species, *Foeniculum vulgare* Mill., but divided into two subspecies, subsp. *vulgare* and subsp. *piperitum* (Ucria) Bég. [33,34].

The Flora Iberica [24] distinguishes subsp. *vulgare* as sweet-fruited, and subsp. *piperitum* as bitter-fruited, although the continued variation in sweet and bitter characters makes this clear division impossible in some cases. The aniseed-like odor and sharp, sweet taste of sweet fennel is ascribed to high levels of *trans*-anethole, some estragole, and fenchone. Bitter fennel, on the other hand, with lower *trans*-anethole and higher estragole and some fenchone content is characterized by a camphorwood flavor and pungent odor [24,35].

In Flora Europaea [25], the Florence fennel, of which the fresh base of the stems is consumed, is the variety *azoricum* [*Foeniculum vulgare* var. *azoricum* (Mill.) Thell.]. The culinary Florence fennel, *F. vulgare* subsp. *vulgare* var. *dulce* is sometimes confused with *F. vulgare* subsp. *vulgare* var. *azoricum*, but it does not have finocchio’s thick leaf stalk base.

The genus shows a high level of morphological variability, and other sources may consider various taxonomic classifications [33,34], but there seems to be consensus on the terminology of bitter fennel and sweet fennel. *European Pharmacopoeia 10* [30] as well as *Portuguese Pharmacopeia IX* [31] assign three monographs to this genus, two to the fruit and another to the EO. One monograph is dedicated to the dried fruit of *F. vulgare* Miller subsp. *vulgare* var. *vulgare*, the bitter fennel, and another is dedicated to the dried fruit of *F. vulgare* Miller subsp. *vulgare* var. *dulce* (Miller) Thell. While these two monographs refer to tests and assays of some compounds of the respective EOs, namely estragole (methyl chavicol), anethole, and fenchone, the third monograph is entirely dedicated to the EO of bitter fennel fruit. In this case, according to *European Pharmacopoeia 10* [30], the EO obtained from the ripe fruit of bitter fennel must have fenchone contents between 12 and 25% and *trans*-anethole contents between 55 and 75%.

ISO 17412:2007 [36] considers two types of bitter fennel EO, the anethole type and the phellandrene type. The available data show the variability in the chemical composition of the fennel EO, which is not yet properly clarified [37].

In an increasingly globalized world, various phenomena of genetic erosion may lead to the loss of autochthonous varieties, which are duly adjusted to local environmental conditions, and may have high potential to produce biomass and bioactive compounds. In this sense, the work of several national entities, from each country, in the preservation and cataloguing of varieties of local ethnobotanical interest is highly important. This type of work involves different approaches which assess discriminating morpho-agronomic, genetic, and traditional knowledge descriptors of each species. Nevertheless, it is not only important to perform agronomic characterization of species relevant to the country but also to complement these data with information on their chemical composition and variability to create a database with ethnobotanical, morphological, and chemical descriptors, as envisaged by Lopes et al. [38].

This work aims to contribute to the knowledge of the chemical variability of Portuguese medicinal and aromatic plant resources and to provide EOs’ chemical descriptors (the main EOs’ representative and characteristic compounds) to add to the existing morpho-agronomic ones (such as plant habit, flower and leaf patterns, and the presence or absence of glands, among others) [39] for better species characterization. To achieve this, two Portuguese Apiaceae species, *Coriandrum sativum* L. and *Foeniculum vulgare* Mill., were assessed. Coriander samples were obtained from landraces collected by the Escola Superior Agrária de Elvas/Instituto Politécnico de Portalegre (ESAE/IPP) during 2002–2011 and from two commercial varieties (Santo and Roma) [40]. The fennel accessions descend from wild plant seeds from collected natural resources by Banco Português de Germoplasma Vegetal (BPGV) [41]. The coriander and fennel were maintained at the experimental fields of IPP and BPGV, respectively. The EOs’ variability was evaluated from 11 samples of *C. sativum* fruits and vegetative aerial parts and from 19 samples of *F. vulgare* fruits of different geographical origins.

## 2. Results

### 2.1. Coriandrum sativum Fruits and Vegetative Aerial Parts—EO Profile and Cluster Analysis

The EOs isolated from *C. sativum* ranged from <0.05% (*v*/*w*) for the three fruit accessions to <0.05–0.1% (*v*/*w*) for the eight vegetative aerial parts’ accessions evaluated, Table 1.

**Figure 1:**
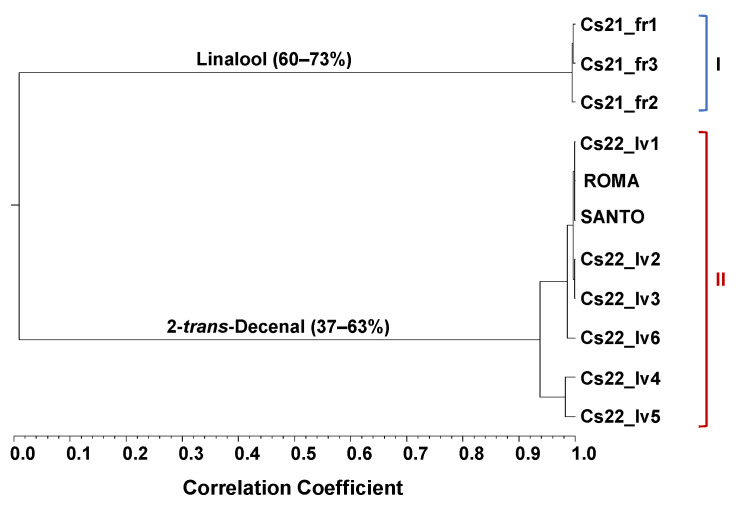
Dendrogram obtained by cluster analysis of the EOs from the 11 *C. sativum* fruit and vegetative aerial parts accessions collected in 2021 and 2022, based on correlation and using the unweighted pair group method with the arithmetic average (UPGMA). For the samples’ codes in each of cluster I and II, see Table 1.

The EOs’ chemical composition showed both qualitative and quantitative differences between the fruits and vegetative aerial parts. Fifty-five compounds were identified in the fruits’ EOs and fifty-eight were identified in the vegetative aerial parts’ EOs, with a percentage of identification >96% in both cases. The relative amounts of all of the identified compounds on each EO accession are listed in Supplementary Material (SM) Table S1. The main identified components (≥2%) are listed in Table 2, following their elution order on the DB-1 column, and they are arranged according to the lowest and the highest percentages found for each component in the two groups defined by agglomerative cluster analysis, based on the chemical composition of all coriander EOs analyzed. This hierarchical clustering highlighted two clusters, clusters I and II, Table 2, with very a low correlation (Scorr < 0.10), as shown in Figure 1.

Cluster I, containing only the three highly correlated (Scorr > 0.95) fruits’ EOs, was dominated by oxygen-containing monoterpenes (66–77%) and monoterpene hydrocarbons (17–22%). The main compounds identified were linalool (60–73%), γ-terpinene (8–12%), followed by α-pinene (2–4%) and geranyl acetate (1–5%) in lower amounts.

Cluster II gathered all the EOs isolated from the vegetative aerial parts, which were also very highly correlated (Scorr > 0.90), showing a dominance of fatty acid derivatives (92–97%), including as the main compounds: 2-*trans*-decenal (37–63%) and *n*-decanal (13–30%) and in lower amounts: 2-*trans*-dodecenal (6–12%), 2-*trans*-undecenal (3–6%), and 2-*trans*-tetradecenal (3–4%). Geranyl acetate (1–5%) and fatty acids like palmitic acid (0.3–2%), myristic acid (0.1–2%), petroselinic acid (t—0.6%), and pentadecanoic acid (t—0.1%) were only identified in the EOs fruit accessions.

### 2.2. Foeniculum vulgare Mill. Fruits—EO Profile and Cluster Analysis

The EOs isolated from the 19 *F. vulgare* fruit accessions were obtained in a yield between 2.6% and 4.8% (*v*/*w*), Table 1. The relative amounts of all identified compounds are given in SM, Table S2. In Table 3, the relative amounts of the main identified components (≥2%) are displayed, according to the minimum and maximum percentage range of components found from each accession in the two groups defined by agglomerative cluster analysis. The chemical composition of the EOs showed a qualitatively similar pattern, where a total of thirty-two compounds were identified, constituting almost 100% of the total composition. The main grouped compounds identified were phenylpropanoids (56–78%) and oxygen-containing monoterpenes (15–35%).

**Figure 2:**
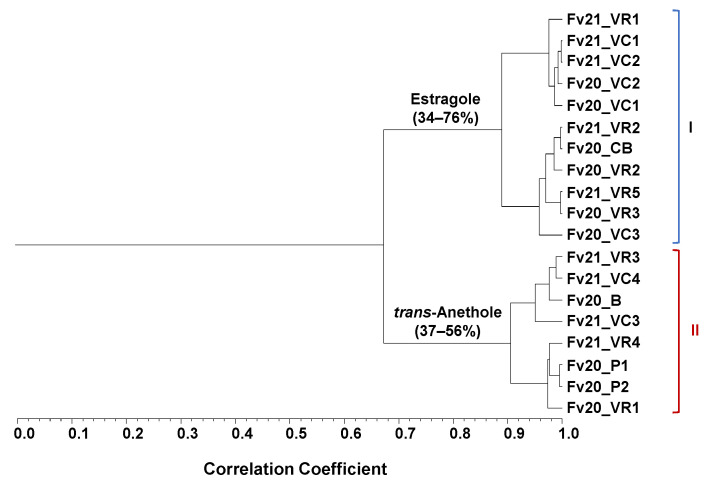
Dendrogram obtained via cluster analysis of the EOs from the 19 *F. vulgare* fruit accessions collected in 2020 and 2021, based on correlation and using the unweighted pair group method with the arithmetic average (UPGMA). For the samples’ codes in each of cluster I and II, see Table 1.

Hierarchical clustering revealed a dendrogram with two clusters moderately correlated (Scorr < 0.68), Figure 2. Cluster I included 11 out of 19 EOs that were dominated by estragole (34–76%), followed by fenchone (16–30%) and *trans*-anethole (1–32%). Cluster II, with eight accessions, showed *trans*-anethole (37–56%), fenchone (14–34%) and the estragole (3–35%) as main EOs’ compounds. Limonene was also identified although in lesser amounts, (1–4%) and (2–7%) in clusters I and II, respectively. The EOs isolated from fruits collected in 2021 and 2022 were evenly distributed over the two clusters. Considering the collection site of the plant material maintained in the experimental field, cluster I included mainly accessions collected in Vila Real and Viana do Castelo, specifically five of each, while the accessions from Portalegre were positioned in cluster II.

## 3. Discussion

### 3.1. Coriandrum sativum EOs Isolated from Fruits and Vegetative Aerial Parts

The EOs isolated from the coriander fruits were compared to those isolated from the vegetative aerial parts, including two commercial samples in the latter case, evidencing, as expected, differences in the identified compounds. The obtained EOs’ yields were very similar between the analyzed samples, although lower than that specified in both Portuguese Pharmacopeia IX [31] and European Pharmacopoeia 10 [30] for coriander fruits’ EOs, ≥0.3%. This fact may be related to the maturity stage of the fruits, as has been reported by Msaada et al. [42], who showed a marked increase in coriander fruits’ essential oil yield during the fruits’ maturation process, as other in Apiaceae.

The fruits’ EOs were dominated by linalool, which is in agreement with other works that described this compound as leading in EOs obtained from coriander fruits from several European, African, and Asian countries, followed by γ-terpinene and α-pinene [9,28,43,44,45,46,47,48]. Likewise, the obtained data agree with those reported for the same type of EOs obtained in Portugal [15,49]. Linalool combines a pleasant aroma, with floral and pleasant odor notes, with several reported biological activities, like antimicrobial, anti-oxidant, antimutagenic, and anti-inflammatory properties, among others [50,51,52].

The EOs isolated from the vegetative aerial parts included different compounds and shared some identical ones, although in different amounts, with those from the fruits’ EOs. As well as in the present work, *n*-decanal, 2-*trans*-decenal, 2-*trans*-tetradecenal, and *n*-dodecanal were previously identified, as the main components, in the EOs of coriander leaves from Pakistan [44], Poland [29], and Kenya [53].

Although the number of samples involved does not allow for substantial conclusions to be drawn, it is worth mentioning that two of the three EOs were isolated from the upper stem leaves (Cs22_lv4 and Cs22_lv5), separate in the dendrogram from those obtained from the basal leaves, in cluster II. It would be interesting to evaluate these findings to explore the observed differences.

The different compounds identified in the fruits and leaves’ EOs will influence the aroma of both. Thus, the predominance of linalool in the fruits’ EOs gives them a sweet, candy-like, and aromatically spicy aroma. The EOs obtained from the leaves, with a predominance of unsaturated aldehydes, ascribes to this part of the plant a fatty, pungent, floral, and spicy aroma [28,48,50].

Several factors can affect EOs’ composition such as the development stage, the environment, and the genetic and culture conditions. With regard to coriander fruits’ EOs, there is a significant change in linalool production during the maturation process of the plant, with lower amounts at an immature stage and higher amounts at intermediate and mature stages [29,42]. In fact, the linalool content depends on the coriander variety being studied; for instance, the var. *vulgare* Alef showed variation of between 64–71%, and in the var. *microcarpum* DC., the linalool amount varied from 42–53% [54]. In the present work, the linalool content did not show wide variation (60–73%), which may indicate that the plants were at the same ontogenetic phase.

Based on the data obtained in the present study, previous unreported work from the authors, and in accordance with the literature, linalool and the aldehyde 2-*trans*-decenal with variable amounts of *n*-decanal are the putative descriptors for Portuguese *C. sativum* fruits and vegetative aerial parts’ EOs, respectively.

### 3.2. Foeniculum vulgare EOs Isolated from Fruits

In 11 of the 19 analyzed EOs, the yields achieved were within the minimum value (≥4%) for bitter fennel, and the remaining yields were above the minimum for sweet fennel (≥2%), in reference to *Portuguese Pharmacopoeia IX* and *European Pharmacopoeia 10* [30,31]. The EO yields achieved are quite desirable since fennel’s biological activity is mostly assigned to its essential oil [55,56].

Since in all EO samples, regardless of the cluster in which they were grouped, the *trans*-anethole content was never ≥80%, the EOs were classified under the generic designation of bitter fennel EO. Furthermore, and considering the classification of ISO 17412:2007 [36], due to the fact that the α-phellandrene content was ≤1.2% and that of limonene was ≤7.1%, Table 3, these EOs may be classified as the bitter fennel anethole type. However, the high content of fenchone in some cases and estragole in others suggests the existence of additional types of bitter fennel EOs of the *trans*-anethole type, with high fenchone and/or estragole content.

Several studies with Portuguese fennel also mention the occurrence of different *trans*-anethole, fenchone, and estragole contents [13,19,20,37,56,57,58,59,60], as observed in the current work. A study on the EO of fennel from Sudan classified it as the estragole chemotype, since this was the dominant compound [61], while Garzoli et al. [18] identified the *o*-cymene chemotype in the EO of fennel from Tarquinia in Italy. Additionally, differences in the composition of fennel EOs from various regions in Tuscany, Italy, and commercial samples have been reported, highlighting differences in *trans*-anethole, fenchone, and estragole content [55].

Since the study of Pujadas Salvà et al. in 2015 [33], the genus *Foeniculum* has changed from being considered monospecific, including just *F. vulgare* Mill., to also include *F. sanguineum* Triano and A. Pujadas. This species shows not only distinctive morphological and molecular characters of *F. vulgare* but also distinct chemical ones. Pujadas Salvà et al. [33] showed that this species fruits’ EOs were characterized by high limonene (60%) and piperitone oxide content (21%), with the later compound not being detected in *F. vulgare* fruits’ EOs. In the current study, the limonene content was always <7%, and piperitone oxide was not detected.

The composition of fennel EO is dependent on several factors such as the ontogenetic phase of the plant, the collection site, the cultivation and storage conditions, and the plant organ from which the EO is obtained, among others [35,62]. Thus, depending on the purpose for which the OE is to be used, it is fundamental to determine its composition in order to meet the requirements of the several target industries, whether food and/or pharmaceuticals [35]. For instance, it has been proposed that a limit of 0.05 mg/kg should be required for the use of estragole in food due to its possible hepatocarcinogenic effect [63].

The present study was run in two consecutive years, both characterized by high temperatures [64]. Nevertheless, to ascertain the effect of increasing temperatures on plant biomass, as well as essential oil yield and composition, a time-course study over more years should be performed. Additionally, the fact that the samples from both 2020 and 2021 were grouped into the same cluster does not allow for a formal conclusion on the influence of increasing temperature on essential oil composition and yield. From data acquired in the current study, as well as in other studies on Portuguese fennel reviewed in [37], *trans*-anethole and estragole, with different amounts of fenchone, comprise the putative descriptors for the Portuguese EOs from the fruits of *F. vulgare.*

## 4. Materials and Methods

### 4.1. Plant Material

**Figure 3:**
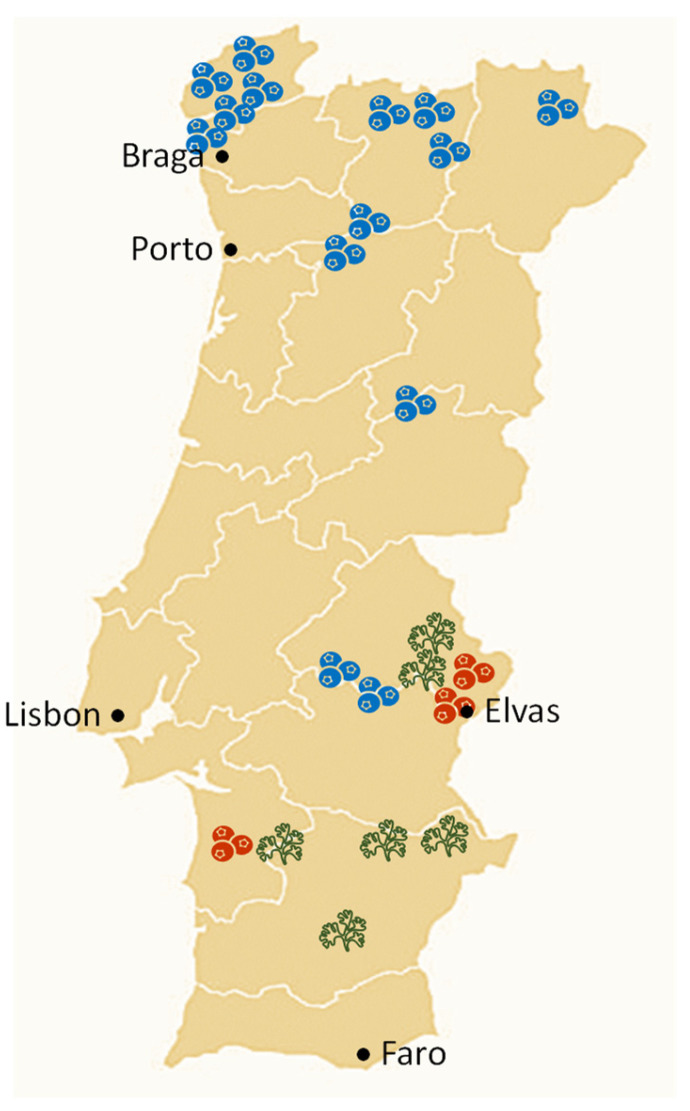
Samples’ collection sites in mainland Portugal. At some collection sites, more than one sample was collected, as detailed in Table 1. 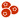 coriander fruits, 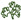 vegetative aerial parts, and 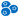 fennel fruits.

The *Coriandrum sativum* fruits and vegetative aerial parts’ accessions under study were cultivated in the experimental fields of Escola Superior Agrária de Elvas/Instituto Politécnico de Portalegre (ESAE/IPP), included in the plant breeding program developed with landraces collected in southern mainland Portugal [65], Table 1. The *Foeniculum vulgare* fruit accessions analyzed in this work were maintained at Banco Português de Germoplasma Vegetal (BPGV)/Instituto Nacional de Investigação Agrária e Veterinária (INIAV). The plant material was obtained for two consecutive years from (a) 3 fruit accessions and 8 vegetative aerial parts’ accessions from *C. sativum* landraces and 2 commercial varieties (ROMA and SANTO) and (b) 19 fruit accessions from *F. vulgare*. The sampling details for each of the analyzed plant materials, and their origin, are provided in Figure 3 and Table 1.

### 4.2. Essential Oils Isolation

Essential oils were obtained by hydrodistillation from coriander fruits and dried vegetative aerial parts and fennel fruits, in a Clevenger-type apparatus according to the *European Pharmacopoeia* [66]. The extraction procedure was performed for 3 h at a distillation rate of 3 mL/min, and the essential oil samples were stored at −20 °C until analysis.

The low-yield EOs were recovered from the Clevenger apparatus graduated tube by rinsing with in-laboratory distilled *n*-pentane when the distillation procedure was over and were allowed to cool (10–15 min). This was accomplished by introducing the distilled *n*-pentane into the filling funnel after flowing out part of the hydrolate of the connecting tube until just below the filling funnel. The residual heat of the distillation flask evaporated the distilled *n*-pentane, which then condensed, and dissolved the essential oil above the aqueous phase in the graduated tube. The mixture of distilled *n*-pentane and essential oil was then recovered in a vial and concentrated to ≈100 µL using a blow-down evaporator system at room temperature under nitrogen flux.

### 4.3. Analysis and Compound Quantification of the EOs

#### 4.3.1. Gas Chromatography with Flame Ionization Detection (GC-FID)

The GC-FID instrument was a PerkinElmer Clarus 400 gas chromatograph (PerkinElmer, Waltham, MA, USA) equipped with two flame ionization detectors with a data handling system. Two columns of different polarities were inserted into the injector port: a DB-1 fused-silica column (100% dimethylpolysiloxane, 30 m × 0.25 mm i.d., film thickness 0.25 µm; J & W Scientific Inc., Folsom, CA, USA) and a DB-17HT fused-silica column ((50% phenyl)-methylpolysiloxane, 30 m × 0.25 mm i.d., film thickness 0.15 µm; J & W Scientific). The initial oven temperature was 45 °C, programmed to rise to 175 °C at 3 °C/min and then from 15 °C/min to 300 °C, where it was held isothermally for 10 min (total run time of 61.67 min). The gas chromatographic settings were as follows: the injector and detector temperatures were 280 °C and 290 °C, respectively, the carrier gas was H_2_ at 30 cm/s, and the split injector ratio was 1:40. The EOs’ percentage composition was determined by integration of the peak areas without the use of correction factors, in accordance with ISO 7609 [67]. The values shown represent the mean value of two injections per sample.

#### 4.3.2. Gas Chromatography–Mass Spectrometry (GC-MS)

The GC-MS unit consisted of a PerkinElmer Clarus 690 gas chromatograph equipped with a DB-1 fused-silica column (100% dimethylpolysiloxane, 30 m × 0.25 mm i.d., film thickness 0.25 µm; J & W Scientific), interfaced with a PerkinElmer SQ 8 T mass spectrometer (software version 6.1, PerkinElmer, Shelton, CT, USA). The injector and oven temperatures were as detailed in Section 4.3.1. The transfer line was at 280 °C, and the ion source was at 220 °C. The carrier gas was helium, adjusted to 30 cm/s. The split ratio was 1:40, and the ionization energy was 70 eV. The scan range was set to 40–300 *m*/*z*, and the scan time was set to 1 s.

Component identification was assigned by comparing their retention indices (RIs), calculated according to ISO 7609 [67], relative to a C_8_–C_23_ *n*-alkane ladder (Sigma) and from mass spectra from a custom-made library based upon commercially available standards (Extrasynthese, Cymit Química, S.L.; Sigma-Aldrich; Fluka, Riedel-de Haën), laboratory-synthesized components [68,69], laboratory isolated compounds [70,71,72,73,74], and reference essential oils of *Thymus caespititius* [75], *Juniperus cedrus* [76], and *Cryptomeria japonica* [77], in which the components’ identity was confirmed by RI, GC-MS, and ^13^C-NMR.

### 4.4. Statistical Analysis

The EOs’ percent composition was used to evaluate the relationship between the various samples through cluster analysis using the Numerical Taxonomy Multivariate Analysis System (NTSYS PC software, version 2.2, Exeter Software, Exeter University, Exeter, UK) [78]. As the agglomerative clustering method, sequential agglomerative hierarchical nested cluster analysis (SAHN) was chosen. The percent composition data matrix was standardized to eliminate the effects of different scales of identification. For the cluster analysis, correlation coefficient was selected as a measure of similarity among all samples, and the unweighted pair group method with arithmetical averages (UPGMA) was used for cluster definition.

The correlation degree was assessed according to Pestana and Gageiro [79] as very high [0.90, 1.00], high [0.70, 0.90[, moderate [0.40, 0.70[, low [0.20, 0.40[, and very low (<0.20).

## 5. Conclusions

Both coriander and fennel are prized species in Portugal for their culinary use, as well as for their traditional medicinal value. Fennel essential oil is also produced in Portugal for diverse food, medicinal, and cosmetics purposes. Given the importance of these species in the national context, it is important to invest in improvement programs, with the view of identifying and selecting lines with higher production and predictable commercial interest, aiming for their registration in the *Catálogo Nacional de Variedades* (CNV, National Variety Catalog) [80] available to producers. Based on the studies thus far, three coriander varieties have been registered in the Portuguese CNV. Medicinal and aromatic plant breeding has its roots in the valorization of natural resources, with local varieties/landraces conserved ex situ in collections in gene banks. In this sense, it is important to obtain the largest set of agronomic and chemical traits, which substantiate the value of the species.

This study allowed for confirmation of the chemical stability of coriander essential oils, dominated by linalool in the fruits and *n*-decanal and 2-*trans*-decenal in the aerial parts. On the other hand, fennel fruits’ essential oils showed variability, suggesting that its descriptors should include fenchone and estragole in addition to *trans*-anethole.

Since both plants, or their essential oils, are used both in food and for cosmetics and medicinal use, it is critical to obtain safe EOs that do not exceed toxic component levels and show high content of compounds of interest. These concerns have a significant economic impact, raising the important issue of the quality control of plant-based products and the need to develop standardized products that are safe for human consumption.

## Figures and Tables

**Table 1:** *Coriandrum sativum* and *Foeniculum vulgare* accessions studied, collection site, plant part studied, plantation and collection dates, EO yields, and accessions codes.

Experimental Field	BPGV Accession	Accession Origin Municipality/District	Plant Material	Plantation Date (Month/Year)	Harvest Date (Month/Year)	EO Yield (%, *v*/*w*)	Accession Code
***Coriandrum sativum***							
ESAE/IPP	BPGV08514	Elvas, Portalegre	Fruits	03/21	06/21	<0.05	Cs21_fr1
ESAE/IPP	BPGV19290	Alcácer do Sal, Setúbal	Fruits	03/21	06/21	<0.05	Cs21_fr2
ESAE/IPP	BPGV28150	Campo Maior, Portalegre	Fruits	03/21	06/21	<0.05	Cs21_fr3
ESAE/IPP	BPGV08514	Elvas, Portalegre	VAPs	02/22	05/22	0.11	Cs22_lv1
ESAE/IPP	BPGV19290	Alcácer do Sal, Setúbal	VAPs	02/22	05/22	0.09	Cs22_lv2
ESAE/IPP	BPGV28150	Campo Maior, Portalegre	VAPs	02/22	05/22	0.06	Cs22_lv3
ESAE/IPP	-	CV	VAPs	02/22	05/22	0.07	ROMA
ESAE/IPP	-	CV	VAPs	02/22	05/22	0.07	SANTO
ESAE/IPP	BPGV19280	Amareleja, Beja	VAPs	02/22	05/22	<0.05	Cs22_lv4
ESAE/IPP	BPGV19282	Castro Verde, Beja	VAPs	02/22	05/22	<0.05	Cs22_lv5
ESAE/IPP	BPGV19284	Vidigueira, Beja	VAPs	02/22	05/22	0.10	Cs22_lv6
***Foeniculum vulgare***							
BPGV	BPGV10429	Avis, Portalegre	Fruits	04/20	10/20	3.00	Fv20_P1
BPGV	BPGV10439	Sousel, Portalegre	Fruits	04/20	10/20	4.00	Fv20_P2
BPGV	BPGV11263	Bragança, Bragança	Fruits	04/20	10/20	4.50	Fv20_B
BPGV	BPGV12149	Vila Real, Vila Real	Fruits	04/20	10/20	4.00	Fv20_VR1
BPGV	BPGV12172	Vila Real, Vila Real	Fruits	04/20	10/20	4.50	Fv20_VR2
BPGV	BPGV12179	Mesão Frio, Vila Real	Fruits	04/20	10/20	4.00	Fv20_VR3
BPGV	BPGV16265	Monção, Viana do Castelo	Fruits	04/20	10/20	4.00	Fv20_VC1
BPGV	BPGV16268	Valença, Viana do Castelo	Fruits	04/20	10/20	3.00	Fv20_VC2
BPGV	BPGV16271	Viana do Castelo, Viana do Castelo	Fruits	04/20	10/20	4.50	Fv20_VC3
BPGV	BPGV16428	Fundão, Castelo Branco	Fruits	04/20	10/20	4.00	Fv20_CB
BPGV	BPGV12198	Montalegre, Vila Real	Fruits	05/21	11/21	2.79	Fv21_VR1
BPGV	BPGV12221	Vila Real, Vila Real	Fruits	05/21	11/21	3.67	Fv21_VR2
BPGV	BPGV12225	Valpaços, Vila Real	Fruits	05/21	11/21	4.69	Fv21_VR3
BPGV	BPGV12231	Chaves, Vila Real	Fruits	05/21	11/21	3.25	Fv21_VR4
BPGV	BPGV12233	Chaves, Vila Real	Fruits	05/21	11/21	4.42	Fv21_VR5
BPGV	BPGV16276	Ponte da Barca, Viana do Castelo	Fruits	05/21	11/21	3.49	Fv21_VC1
BPGV	BPGV16279	Paredes de Coura, Viana do Castelo	Fruits	05/21	11/21	4.80	Fv21_VC2
BPGV	BPGV16285	Ponte da Barca, Viana do Castelo	Fruits	05/21	11/21	2.58	Fv21_VC3
BPGV	BPGV16294	Ponte de Lima, Viana do Castelo	Fruits	05/21	11/21	2.88	Fv21_VC4

ESAE/IPP: Escola Superior Agrária de Elvas/Instituto Politécnico de Portalegre. BPGV: Banco Português de Germoplasma Vegetal. VAPs: vegetative aerial parts. CV: commercial variety.

**Table 2:** Minimum and maximum percent composition range of the EOs’ main compounds (≥2% in at least one sample) isolated from three fruit accessions and eight vegetative aerial parts accessions of *C. sativum*. For the samples grouped in clusters I and II, see Figure 1. The complete and detailed composition is shown in SM Table S1.

Components	RI	Cluster I		Cluster II	
		Min	Max	Min	Max
*n*-Nonane	900	t	t	0.3	3.1
α-Pinene	930	1.7	4.3	t	0.3
γ-Terpinene	1035	8.1	12.0	t	0.3
Linalool	1074	**59.6**	**72.6**	t	0.4
*n*-Decanal	1180	0.7	1.9	**13.2**	**30.2**
2-*trans*-Decenal	1236	1.4	3.3	**36.7**	**63.3**
2-*trans*-Undecenal	1334	t	0.1	2.6	6.2
Geranyl acetate	1370	1.4	4.5		
2-*trans*-Dodecenal	1446	0.3	1.0	6.0	11.8
2-*trans*-Tetradecenal *	1643	0.1	0.2	2.5	4.1
**% Identification**		98.9	99.7	96.9	98.3
**Grouped components**					
Monoterpene hydrocarbons		16.9	22.0	0.6	1.3
Oxygen-containing monoterpenes		66.3	76.7	0.1	0.5
Sesquiterpene hydrocarbons		t	0.1	t	t
Oxygen-containing sesquiterpenes		t	0.3	t	0.1
Oxygen-containing diterpenes				0.2	0.4
Fatty acids		0.4	4.8		
Fatty acid derivatives		4.0	9.2	91.8	96.6
Others		t	t	0.4	3.5

RI: in-lab calculated retention index relative to C_8_–C_23_ *n*-alkanes on the DB-1 column. Min: minimum. Max: maximum. t: traces (<0.05%). * Identification based on mass spectrum only. Bold: dominant compounds relevant to each cluster.

**Table 3:** Minimum and maximum percent composition range of the EOs’ main compounds (≥2% in at least one sample) isolated from 19 fruit accessions of *F. vulgare*. For the samples grouped into clusters I and II, see Figure 2. The complete and detailed composition is shown in SM Table S2.

Components	RI	Cluster I		Cluster II	
		Min	Max	Min	Max
α-Pinene	930	0.4	2.7	0.5	1.2
β-Myrcene	975	0.4	1.5	0.7	1.5
1,8-Cineole	1005	0.3	1.6	0.3	2.0
Limonene	1009	1.3	3.5	2.2	7.1
γ-Terpinene	1035	0.3	2.4	0.3	2.2
Fenchone	1050	**15.8**	**29.6**	**13.6**	**34.1**
Estragole (=Methyl chavicol)	1163	**34.0**	**75.5**	3.2	35.1
*trans*-Anethole	1254	1.0	32.0	**37.0**	**56.3**
**% Identification**		99.8	99.9	99.8	99.9
**Grouped components**					
Monoterpene hydrocarbons		3.5	9.4	6.6	13.2
Oxygen-containing monoterpenes		16.7	31.3	15.1	35.3
Phenylpropanoids		62.2	77.9	56.3	75.2
Others		t	t	t	t

RI: in-lab calculated retention index relative to C_9_–C_20_ *n*-alkanes on the DB-1 column. Min: minimum. Max: maximum. t: traces (<0.05%). Bold: dominant compounds relevant to each cluster.

## Data Availability

Not applicable.

## Supplementary Materials

The following supporting information can be downloaded at: https://www.mdpi.com/article/10.3390/plants12142749/s1. Table S1. Percentage composition of the EOs isolated by hydrodistillation from *Coriandrum sativum* fruits and vegetative aerial parts’ accessions grown in the experimental field of Escola Superior Agrária de Elvas. For the samples’ codes, see Table 1; Table S2. Percentage composition of the EOs isolated by hydrodistillation from *Foeniculum vulgare* fruit accessions grown in the experimental field of Banco Português de Germoplasma Vegetal. For the samples’ codes, see Table 1.
