# Supplementary material for: Chemical Variability of the Essential Oils from Two Portuguese Apiaceae: Coriandrum sativum L. and Foeniculum vulgare Mill
Source: Plants (Basel). 2023 Jul 24;12(14):2749. doi: 10.3390/plants12142749 (PMC10384636; doi:10.3390/plants12142749)
Supplement: Supplementary file 1 [file plants-12-02749-s001.zip › plants-2516984-supplementary.pdf]

plants

MDPI

Article

Chemical variability of the essential oils from two Portuguese Apiaceae: *Coriandrum sativum* L. and *Foeniculum vulgare* Mill.

Alexandra M. Machado [1,*], Violeta Lopes [2], Ana M. Barata [2], Orlanda Póvoa [3,4], Noémia Farinha [4] and A. Cristina Figueiredo [1,*]

1 Centro de Estudos do Ambiente e do Mar (CESAM Lisboa), Faculdade de Ciências da Universidade de Lisboa (FCUL), Biotecnologia Vegetal, DBV, C2, Campo Grande, 1749-016 Lisboa, Portugal
2 Banco Português de Germoplasma Vegetal (BPGV), Instituto Nacional de Investigação Agrária e Veterinária, Quinta de S. José, S. Pedro de Merelim, 4700-859 Braga, Portugal; violeta.lopes@iniav.pt (V. L.); anamaria.barata@iniav.pt (A.M.B.)
3 VALORIZA—Centro de Investigação para a Valorização de Recursos Endógenos, Instituto Politécnico de Portalegre, Praça do Município 11, 7300-110 Portalegre, Portugal; opovoa@ipportalegre.pt
4 Instituto Politécnico de Portalegre, Praça do Município 11, 7300-110 Portalegre, Portugal; nfarinha@ipportalegre.pt
* Correspondence: ialexam@gmail.com (A.M.M.); acsf@fc.ul.pt (A.C.F.); Tel.: +351-21-750-0257 (A.M.M. & A.C.F.)

Table S1. Percentage composition of the EOs isolated by hydrodistillation, from *Coriandrum sativum* fruits and vegetative aerial parts accessions grown at the experimental field of Escola Superior Agrária de Elvas. For samples codes, see Table 1.

Components	RI	Accessions										
		Fruits			Vegetative aerial parts							
		Cs21_fr1 2021	Cs21_fr2 2021	Cs21_fr3 2021	Cs22_lv1 2022	Cs22_lv2 2022	Cs22_lv3 2022	ROMA 2022	SANTO 2022	Cs22_lv4 2022	Cs22_lv5 2022	Cs22_lv6 2022
n-Octane	800				t	t	t	t	t	t	t	t
2-*trans*-Hexenal	866				0.1	0.2	0.1	0.1	0.1	0.1	t	0.2
cis-2-Hexen-1-ol	882				t	t	t	t	t	t	t	t
n-Hexanol	883				t	t	t	t	t	t	t	t
n-Heptanal	897	t	0.1	0.1	t	t	t	t	t	t	t	t
1-Nonene	899				t	t	t	t	t	t	t	t
n-Nonane	900	t	t	t	2.1	2.9	2.8	3.1	1.9	0.3	0.7	1.8
trans-3-Nonene *	901				t	t	t	t	t	t	t	t
Tricyclene	921	t	t	t								
α-Thujene	924	t	t	0.1								
α-Pinene	930	1.7	4.3	3.6	t	t	0.1	0.1	0.1	t	t	0.3
Camphene	938	0.1	0.4	0.3								
n-Heptanol	952	t	t	t	t	t	t	t	t	t	t	t
Sabinene	958	0.2	0.4	0.4	t	t	t	t	t	t	t	t
β-Pinene	963	0.3	0.5	0.5	t	t	t	t	t	t	t	t
3-Methyl nonane *	970				t	t	t	t	t	t	t	t
2-Pentyl furan	973	t	t	t								
n-Octanal	973	0.1	t	0.1	1.1	1.1	1.2	1.2	1.2	1.1	0.7	0.8
β-Myrcene	975	0.7	1.1	0.9	0.4	0.4	0.5	0.7	0.7	0.5	0.5	0.5
α-Phellandrene	995	t	t	t								
n-Decane	1000	t	t	t	0.1	0.2	0.2	0.2	0.1	t	0.1	0.1
Phenylacetaldehyde (Benzene acetaldehyde)	1002				t	t	t	t	t	t	t	t
α-Terpinene	1002	0.1	0.1	0.1								
p-Cymene	1003	1.6	1.4	2.1	t	t	t	t	t	t	t	t
1,8-Cineole	1005	t	t	t	t	t	t	t	t	0.1	0.1	t

**Components**	**RI**	**Accessions**										
		**Fruits**			**Vegetative aerial parts**							
		**Cs21_fr1 2021**	**Cs21_fr2 2021**	**Cs21_fr3 2021**	**Cs22_lv1 2022**	**Cs22_lv2 2022**	**Cs22_lv3 2022**	**ROMA 2022**	**SANTO 2022**	**Cs22_lv4 2022**	**Cs22_lv5 2022**	**Cs22_lv6 2022**
β-Phellandrene	1005	0.1	0.1	0.1								
Limonene	1009	1.2	1.8	1.4	0.2	0.3	0.4	0.3	0.1	0.3	0.3	0.1
cis-β-Ocimene	1017	t	t	t								
trans-β-Ocimene	1027	0.1	t	0.1	t	t	t	t	t	t	t	0.1
γ-Terpinene	1035	10.4	8.1	12.0	t	t	t	t	t	t	t	0.3
trans-Sabinene hydrate	1037	t	t	t								
n-Octanol	1045	0.2	t	0.1	t	t	t	t	t	t	0.1	t
Terpinolene	1064	0.4	0.4	0.4	t	t	t	t	t	t	t	t
n-Nonanal	1073	1.1	0.4	0.4	0.6	0.7	1.0	0.6	0.6	0.4	0.6	0.5
Linalool	1074	**64.0**	**72.6**	**59.6**	0.1	0.2	0.1	0.1	0.1	t	t	0.4
n-Undecane	1100				t	t	t	0.1	t	t	t	t
Camphor	1102	1.3	2.1	0.9	t	t	t	t	t	t	t	t
Isopulegol	1121	0.1	0.1	t								
2-*trans*-Nonen-1-al	1124				t	0.1	0.1	0.1	0.1	0.1	t	t
Borneol	1134	t	t	t								
n-Nonanol	1148				0.1	0.1	0.1	0.1	0.1	t	t	t
Terpinen-4-ol	1148	0.2	0.2	0.1								
α-Terpineol	1159	0.1	0.2	0.1								
4-*cis*-Decenal	1163				0.7	1.1	1.0	1.1	1.1	1.0	1.1	0.6
n-Decanal	1180	1.9	0.7	1.6	**30.2**	**28.1**	**26.2**	**29.4**	**29.3**	**13.2**	**19.3**	**23.5**
Pulegone	1207				t	t	t	t	t	t	t	t
Citronellol	1211	0.2	0.1	0.1								
Geraniol	1236	0.8	t	0.9								
2-*trans*-Decenal	1236	3.3	1.9	1.4	**40.1**	**40.7**	**39.2**	**36.7**	**38.0**	**63.3**	**49.3**	**39.1**
2-*trans*-Decen-1-ol *	1256				1.0	1.2	1.7	1.8	1.7	0.6	0.7	0.5
n-Decanol	1259	0.2	0.1	0.4	1.3	1.3	1.7	1.7	1.8	0.6	0.5	0.3
p-Cymen-7-ol	1265	0.1	t	t								
4-Vinylguaiacol	1285				0.1	0.1	0.1	0.1	0.1	t	t	0.1

Components	RI	Accessions										
		Fruits			Vegetative aerial parts							
		**Cs21_fr1 2021**	**Cs21_fr2 2021**	**Cs21_fr3 2021**	**Cs22_lv1 2022**	**Cs22_lv2 2022**	**Cs22_lv3 2022**	**ROMA 2022**	**SANTO 2022**	**Cs22_lv4 2022**	**Cs22_lv5 2022**	**Cs22_lv6 2022**
n-Undecanal	1288	0.7	0.3	0.5	1.6	2.0	2.2	1.7	1.6	0.6	1.7	1.9
Myrtenyl acetate	1290	t	t	0.1								
2-*trans*-Undecenal	1334	0.1	t	0.1	3.7	4.0	5.0	2.6	3.0	4.4	6.2	6.0
trans-2-Undecen-1-ol	1355				0.1	0.2	0.3	0.2	0.2	t	0.1	t
1-Undecanol	1366				0.7	0.1	0.2	0.2	0.2	t	0.1	t
Geranyl acetate	1370	1.7	1.4	4.5								
n-Dodecanal	1397	0.3	0.1	0.2	1.6	1.4	1.7	1.9	1.8	1.4	1.9	2.4
β-Caryophyllene	1414	0.1	t	t								
2-*trans*-Dodecenal	1446	1.0	0.3	0.6	7.0	6.8	6.3	6.8	7.4	6.0	8.2	11.8
2-*trans*-Dodecen-1-ol	1448				0.1	0.1	0.2	0.2	0.2	0.1	0.1	0.1
1-Dodecanol	1468				t	t	t	t	t	t	t	t
α-Amorphene	1476				t	t	t	t	t	t	t	t
n-Tridecanal	1499	0.1	t	t	0.1	0.1	0.1	0.1	0.1	0.1	0.2	0.2
trans-Nerolidol	1549	t	t	0.1								
2-*trans*-Tridecen-1-al	1550				0.3	0.3	0.5	0.4	0.4	0.2	0.3	0.6
β-Caryophyllene oxide	1561	0.3	t	t	t	t	t	t	t	t	t	t
n-Tetradecanal	1596	t	t	t	0.3	0.2	0.3	0.3	0.3	0.4	0.4	0.5
T-Cadinol	1616				t	0.1	0.1	0.1	0.1	t	t	t
α-Cadinol	1630				t	t	t	t	t	t	t	t
2-*trans*-Tetradecenal*	1643	0.2	0.1	0.2	3.2	2.5	3.0	4.1	4.0	2.5	2.8	3.7
Pentadecanal	1688				t	t	t	t	t	t	t	t
Tetradecanoic acid (Myristic acid)	1723	2.2	0.1	1.6								
11-Pentadecenal *	1735				0.3	0.3	0.2	0.4	0.4	0.2	0.3	0.4
Pentadecanoic acid *	1776	0.1	t	0.1								
Palmitic acid (Hexadecanoic acid)	1908	1.5	0.3	2.3								
Phytol acetate 2	2101				0.2	0.2	0.3	0.2	0.2	0.4	0.4	0.3
n-Nonadecanal *	2102				0.2	0.2	0.3	0.2	0.2	0.4	0.4	0.3
Linoleic acid	2108	t	t	0.2								

**Components**	**RI**	**Accessions**										
		**Fruits**			**Vegetative aerial parts**							
		**Cs21_fr1 2021**	**Cs21_fr2 2021**	**Cs21_fr3 2021**	**Cs22_lv1 2022**	**Cs22_lv2 2022**	**Cs22_lv3 2022**	**ROMA 2022**	**SANTO 2022**	**Cs22_lv4 2022**	**Cs22_lv5 2022**	**Cs22_lv6 2022**
Petroselinic acid	2128	0.1	t	0.6								
Eicosanal	2200				t	t	t	t	t	t	t	t
**% Identification**		98.9	99.7	98.9	97.6	97.2	97.2	96.9	97.2	98.3	97.1	97.4
**Grouped components**												
Monoterpene hydrocarbons		16.9	18.6	22.0	0.6	0.7	1.0	1.1	0.9	0.8	0.8	1.3
Oxygen-containing monoterpenes		68.8	76.8	66.5	0.2	0.3	0.2	0.2	0.2	0.1	0.1	0.5
Sesquiterpene hydrocarbons		0.1	t	t	t	t	t	t	t	t	t	t
Oxygen-containing sesquiterpenes		0.3	t	0.1	t	0.1	0.1	0.1	0.1	t	t	t
Oxygen-containing diterpenes					0.2	0.2	0.3	0.2	0.2	0.4	0.4	0.3
Fatty acids		3.9	0.4	4.8								
Other fatty acid derivatives					94.3	92.6	92.5	91.8	93.7	96.6	95.0	93.2
Others		8.9	3.9	5.5	2.3	3.3	3.1	3.5	2.1	0.4	0.8	2.1

RI: In-lab calculated retention index relative to C_8-C_{23} *n*-alkanes on the DB-1 column. t: traces (t < 0.05%). * Identification based on mass spectrum only. Bold: dominant compounds relevant for each cluster.

Table S2. Percentage composition of the EOs isolated by hydrodistillation, from *Foeniculum vulgare* fruit accessions grown at the experimental field of Banco Português de Germoplasma Vegetal. For samples codes, see Table 1.

Components	RI	Accessions																		
		**Fv20 _P1 2020**	**Fv20 _P2 2020**	**Fv20 _B 2020**	**Fv20 _VR1 2020**	**Fv20 _VR2 2020**	**Fv20 _VR3 2020**	**Fv20 _VC1 2020**	**Fv20 _VC2 2020**	**Fv20 _VC3 2020**	**Fv20 _CB 2020**	**Fv21 _VR1 2021**	**Fv21 _VR2 2021**	**Fv21 _VR3 2021**	**Fv21 _VR4 2021**	**Fv21 _VR5 2021**	**Fv21 _VC1 2021**	**Fv21 _VC2 2021**	**Fv21 _VC3 2021**	**Fv21 _VC4 2021**
α-Thujene	924	t	t	t	t	t	t	t	t	t	t	t	t	t	t	t	t	t	t	t
α-Pinene	930	0.7	0.6	0.7	0.9	0.8	1.6	0.4	0.4	0.7	0.9	2.7	1.3	1.0	1.2	1.1	2.1	1.1	0.5	1.1
Camphene	938	0.3	0.3	0.2	0.3	0.1	0.3	0.2	0.1	0.1	0.3	0.1	0.2	0.2	0.4	0.3	0.3	0.3	0.2	0.3
Sabinene	958	0.3	0.2	0.3	0.2	0.1	0.5	0.2	0.2	0.1	0.4	0.3	0.5	0.6	0.6	0.6	0.4	0.4	0.6	0.3
β-Pinene	963	0.1	0.1	0.1	0.2	0.1	0.2	0.5	t	0.1	0.1	0.4	t	0.5	0.1	0.1	0.3	0.3	0.4	0.2
β-Myrcene	975	0.9	1.3	0.7	1.0	1.1	1.5	1.0	0.6	0.4	1.3	0.6	0.9	0.8	1.2	1.4	1.2	0.9	1.5	1.0
α-Phellandrene	995	0.3	0.2	0.4	0.4	0.3	0.3	0.4	0.3	0.2	0.3	0.4	0.6	0.4	0.8	0.5	0.8	0.5	1.2	0.6
α-Terpinene	1002	t	t	t	t	t	t	t	t	t	t	t	t	t	t	t	t	t	t	t
p-Cymene	1003	0.1	t	0.1	0.2	t	0.1	0.1	0.1	0.1	0.2	0.1	t	0.2	t	0.2	0.1	t	0.1	0.1
1,8-Cineole	1005	0.8	0.3	0.7	0.7	1.0	1.6	0.5	0.8	0.3	1.2	0.6	1.1	1.0	2.0	1.6	t	1.1	1.3	1.0
β-Phellandrene	1005	t	t	t	t	t	t	t	t	t	t	t	t	t	t	t	1.1	t	t	t
Limonene	1009	3.6	4.9	3.1	3.4	3.5	3.3	2.8	1.4	1.9	2.5	1.3	2.1	7.1	5.1	2.6	2.2	2.7	3.8	2.2
cis-β-Ocimene	1017	t	t	t	0.1	t	0.1	t	t	t	0.1	t	t	t	t	t	t	t	t	t
trans-β-Ocimene	1027	t	t	t	t	t	t	t	t	t	t	t	t	t	t	t	t	t	t	t
γ-Terpinene	1035	1.0	0.4	0.9	1.1	0.3	1.0	1.1	0.3	0.4	1.6	0.8	0.5	2.2	0.3	2.4	0.7	0.6	1.3	0.9
trans-Sabinene hydrate	1037	0.2	0.1	0.1	0.1	0.1	0.1	0.1	0.1	0.1	0.1	t	0.1	0.2	0.1	0.2	0.1	t	t	0.1
Fenchone	1050	**32.1**	**34.1**	**21.0**	**22.5**	**29.6**	**22.5**	**26.2**	**17.5**	**17.3**	**25.9**	**15.8**	**26.8**	**23.1**	**28.9**	**24.4**	**17.8**	**19.2**	**13.6**	**27.3**
6,7-Myrcene epoxide	1064											t	t	t	t	t	t	t	t	t
Terpinolene	1064	0.2	0.3	0.1	0.1	0.1	0.2	0.3	0.1	0.1	0.2	t	0.1	0.2	0.2	0.2	0.1	0.1	t	0.2
cis-Sabinene hydrate	1066	t	t	t	t	t	t	t	t	t	t	t	t	t	t	t	t	t	t	t
Linalool	1074											t	0.4	t	0.1	t	t			t
Isopentyl isovalerate	1080											t		t	t	t	t	t	t	t
α-Fenchol (*endo*-Fenchol)	1085											t	t	0.2	t	0.2	t	t	t	t
Camphor	1102	0.7	0.7	0.4	0.5	0.6	0.5	0.5	0.4	0.2	0.5	0.3	0.5	0.5	0.5	0.5	0.3	0.4	0.2	0.5
δ-Terpineol	1134												t	t	t	t		t		
Terpinen-4-ol	1148	0.1	0.1	t	t	t	0.1	t	0.1	t	0.1	t	t	0.1	t	0.1	t	t	t	t

Components	RI	Accessions																		
		**Fv20_P1 2020**	**Fv20_P2 2020**	**Fv20_B 2020**	**Fv20_VR1 2020**	**Fv20_VR2 2020**	**Fv20_VR3 2020**	**Fv20_VC1 2020**	**Fv20_VC2 2020**	**Fv20_VC3 2020**	**Fv20_CB 2020**	**Fv21_VR1 2021**	**Fv21_VR2 2021**	**Fv21_VR3 2021**	**Fv21_VR4 2021**	**Fv21_VR5 2021**	**Fv21_VC1 2021**	**Fv21_VC2 2021**	**Fv21_VC3 2021**	**Fv21_VC4 2021**
α-Terpineol	1159	t	t	t	t	t	t	t	t	t	t	t	0.1	0.1	0.1	0.2	t		t	t
Estragole (Methyl chavicol)	1163	**3.2**	**6.2**	**22.5**	**11.9**	**37.0**	**34.0**	**56.7**	**68.5**	**51.7**	**45.0**	**75.5**	**42.3**	**23.1**	**14.8**	**34.1**	**59.4**	**57.5**	**35.1**	**27.1**
p-Anisaldehyde	1210	t	t	t	t	t	t	t	t	t	t	t	t	t	t	t	t	t	t	t
cis-Anethole	1220	t	t	t	t	t	t	t	t	t	t	t	t	t	t	t	t	t	t	t
trans-Anethole	1254	**55.3**	**50.1**	**48.5**	**56.3**	**25.2**	**32.0**	**8.9**	**8.9**	**26.2**	**19.2**	**1.0**	**22.4**	**38.4**	**43.5**	**29.2**	**13.0**	**14.8**	**40.1**	**37.0**
Palmitic acid (Hexadecanoic acid)	1908	t	t	t	t	t	t	t	t	t	t									
**% Identification**		99.9	99.9	99.8	99.9	99.9	99.9	99.9	99.8	99.9	99.9	99.9	99.9	99.9	99.9	99.9	99.9	99.9	99.9	99.9
**Grouped components**																				
Monoterpene hydrocarbons		7.5	8.3	6.6	7.9	6.4	9.1	7.0	3.5	4.1	7.9	6.7	6.2	13.2	9.9	9.4	9.3	6.9	9.6	6.9
Oxygen-containing monoterpenes		33.9	35.3	22.2	23.8	31.3	24.8	27.3	18.9	17.9	27.8	16.7	29.0	25.2	31.7	27.2	18.2	20.7	15.1	28.9
Phenylpropanoids		58.5	56.3	71.0	68.2	62.2	66.0	65.6	77.4	77.9	64.2	76.5	64.7	61.5	58.3	63.3	72.4	72.3	75.2	64.1
Others		t	t	t	t	t	t	t	t	t	t									

RI: In-lab calculated retention index relative to C_9-C_{20} *n*-alkanes on the DB-1 column. t: traces (t < 0.05%). Bold: dominant compounds relevant for each cluster.
